# Effects of Dietary Lysine Level on Growth Performance and Protein Metabolism in Juvenile Leopard Coral Grouper (*Plectropomus leopardus*)

**DOI:** 10.1155/2023/1017222

**Published:** 2023-06-03

**Authors:** Xiaomei Dou, Yu Liu, Yixiong Cao, Yumeng Zhang, Xinlangji Fu, Junming Deng, Beiping Tan

**Affiliations:** ^1^College of Fisheries, Guangdong Ocean University, Zhanjiang 524088, China; ^2^Guangdong Engineering Technology Research Center of Aquatic Animals Precision Nutrition and High Efficiency Feed, Zhanjiang 524088, China; ^3^Key Laboratory of Aquatic, Livestock and Poultry Feed Science and Technology in South China, Ministry of Agriculture, Zhanjiang 524088, China

## Abstract

An 8-week feeding trial was conducted to evaluate the effects of dietary lysine level on growth performance and protein metabolism of juvenile leopard coral grouper (*Plectropomus leopardus*) and thereby obtained the optimal dietary lysine requirement of *P. leopardus*. Six isoproteic and isolipidic experimental diets were formulated to contain 1.10%, 1.69%, 2.30%, 3.08%, 3.56%, and 4.36% lysine of diets, respectively. Each diet was assigned at random to triplicate groups of 25 juveniles (initial mean weight is 10.57 g) per tank in a flow-through mariculture system maintained at 27–30°C. Dietary inclusion of 2.30–3.08% lysine improved the weight gain rate (WGR) and specific growth rate and decreased the feed conversion ratio (FCR) of juveniles (*P* < 0.05). The intestinal digestive enzyme (trypsin, amylase, and lipase) activities were overall enhanced by dietary inclusion of 3.08–3.56% lysine (*P* < 0.05). The mammalian target of rapamycin (mTOR) signaling pathway was activated in fish fed diets with 1.69–2.30% lysine by upregulating the relative expression levels of hepatic TOR and S6K1 (p70 ribosomal protein S6 kinase 1) but downregulating the relative expression level of hepatic 4E-BP2 (eIF4E-binding protein 2). Conversely, the amino acid response signaling pathway was inhibited in fish fed diet with 2.30% lysine by downregulating the relative expression levels of hepatic GCN2 (general control nondepressible 2), ATF3 (activating transcription factor 3), ATF4a (activating transcription factor 4a), and ATF4b (activating transcription factor 4b). Additionally, dietary 1.69–3.08% lysine enhanced the plasma total protein level and hepatic lysine *α*-ketoglutarate reductase activity but depressed the blood urea nitrogen level and hepatic adenosine monophosphate deaminase activity (*P* < 0.05). Moreover, dietary 3.08% lysine increased the contents of whole-body crude protein and total amino acids, while 1.69%–4.36% lysine depressed the whole-body lipid content (*P* < 0.05). These results indicated that optimal dietary lysine increased the digestive enzyme activities, promoted protein synthesis but depressed protein degradation, and thereby improved the growth performance of *P. leopardus*. Based on the second-order polynomial model, the optimal lysine requirement of juvenile *P. leopardus* for WGR, FCR, and lysine deposition was 2.60%–2.97% of diets (4.91%–5.60% of dietary protein).

## 1. Introduction

Leopard coral grouper *Plectropomus leopardus* (Lacepède, 1802), a top carnivore in many coral reef communities, is mainly distributed from the Western Pacific to East Africa and the Red Sea [1]. Due to its good flavor and bright body color, *P. leopardus* is popular and marketable in China. The decline of wild resources and relatively slow growth rate have led to the increasing price of *P. leopardus*. Thus, this species has become one of the vital breeding varieties of grouper with a broad market prospect [2]. However, there is no specialized compound feed for *P. leopardus* due to the limited information on nutritional requirements [3]. So far, research on the requirement of essential amino acids of *P. leopardus* has not been reported.

Lysine is an alkaline amino acid with little content in cereals. It is an essential amino acid for fish and one of the most important limiting amino acids for most plant ingredients [4]. Decreased feeding, stunted growth, and reduced feed efficiency are the most responsive and apparent symptoms of lysine deficiency in fish. However, excess lysine also has negative effects on growth rate and feed utilization of fish [5, 6] as it disrupts the balance of amino acids in diets and prevents the absorption and utilization of other amino acids [7]. Consequently, it is essential to determine the dietary lysine requirement of fish. To date, there is no research about the requirement of lysine for juvenile *P. leopardus*. Therefore, the current study was designed to assess the effects of dietary lysine level on the growth performance and protein metabolism of juvenile *P. leopardus* and thereby obtained the optimum dietary lysine requirement of *P. leopardus*.

## 2. Materials and Methods

### 2.1. Animal Ethic Statement

All experiments were instructed under the Guidance of the Care and Use of Laboratory Animals in China (GB/T 35892-2018). This research was approved by the Animal Care and Use Committee of Guangdong Ocean University (GDOU-IACUC-2021-A0204).

### 2.2. Experimental Diets

Six isoproteic (53% crude protein) and isolipidic (12% crude lipid) experimental diets were formulated to contain 1.10%, 1.69%, 2.30%, 3.08%, 3.56%, and 4.36% lysine of diet at the expense of alanine, respectively (Table 1). The compositions of the essential amino acid (except for lysine) in the experimental diets were adjusted to the reference amino acid profile established by the previous study [8]. The proximate and amino acid compositions of the experimental diets are present in Tables 1 and 2, respectively.

All separately crushed ingredients (except for fish oil and soybean oil) were sieved through a sixty-mesh sieve and mixed thoroughly. After fish oil and soybean oil (soybean lecithin was predissolved in soybean oil) were added, all ingredients were remixed by a V-type vertical mixer (VI-1000L; Jiake Machinery Manufacturing Co., Ltd., Jiangyin, China). A suitable amount of distilled water (about 20–30%) was added to form a dough, and the dough was processed into 2 mm diameter pellets by a twin-screw extruder (F-26; South China University of Technology, Guangzhou, China). All diets were air-dried at 25°C for 24 h and stored at −20°C until used.

### 2.3. Feeding Management

Juvenile *P. leopardus* were obtained from a local commercial hatchery. After two weeks of cultivation in 1 m^3^ tank, healthy and uniform juveniles (initial mean weight 10.57 ± 0.02 g) were randomly divided into 18 tanks (three replicates per group) with 25 fish per tank (0.3 m^3^). All fish were fed twice daily (8:00 and 16:00) to apparent satiation (about 3% of body weight) and kept under natural photoperiod and continuous aeration. During the experimental period, the water temperature was maintained at 27−30°C, pH 7.7−8.0, ammonia nitrogen < 0.2 mg/L, and dissolved oxygen > 5.0 mg/L. The feeding trial lasted for 8 weeks.

### 2.4. Sample Collection

At the end of the feeding trial, all fish were fasted for 24 hours and anesthetized with MS-222 (Sigma-Aldrich Co., St. Louis, MO, USA). The final numbers and biomass of fish per tank were measured. Three fish per tank were randomly selected and stored at –20°C for analysis of body composition. Another four fish per tank were collected to obtain the weight of the whole body, viscera, liver, and intestine. Blood samples were taken from the caudal vein of six fish per tank with a heparinized syringe and then collected into a heparinized tube; the plasma was collected and stored at –80°C. Four fish per tank were aseptically sacrificed in an ice bath; then, the liver, midgut, and dorsal muscle samples were rapidly removed and frozen in liquid nitrogen and then stored at –80°C until used.

### 2.5. Analysis

#### 2.5.1. Proximate Composition

Proximate composition of the experimental diets and whole-body samples was performed by the following AOAC method: moisture was dried in an oven at 105°C to constant weight; crude protein (nitrogen × 6.25) was determined by the regular Kjeldahl method using Dumas nitrogen analyzer (Primacs SN100; Skalar, Netherlands); crude lipid was reflux extracted with petroleum ether and calculated as the difference between weight of sample and residue; crude ash was incinerated at 550°C in a muffle furnace (SX-410; Beijing Ever Bright Medical Treatment Instrument Co., Ltd., Beijing, China) for 16 h. The amino acid profiles were measured using an automatic amino acid analyzer (Hitachi L8900; Hitachi, Tokyo, Japan) after acid hydrolysis using 6 N HCl at 110°C for 22 h.

#### 2.5.2. Digestive Enzyme Activities

To obtain an adequate crude enzyme extract solution, the amount of physiological saline solution (0.9% NaCl) added to the wet midgut was determined by a preliminary study. The wet midgut plus fourfold volume (*v*/*w*) of ice-cold physiological saline solution was added to a 10 mL test tube and homogenized using an IKA homogenizer (IKA Works Asia, Bhd, Malaysia). The homogenate was centrifuged at 9 000 g for 30 min at 4°C using a high-speed refrigerated centrifuge (MX-160; Tomy Seiko Co., Ltd., Tokyo, Japan). The supernatant was diluted with physiological saline solution in a certain dilution ratio and used as a crude enzyme solution. The activities of intestinal trypsin, lipase, and amylase were detected by the UV colorimetric method, microplate method, and iodine-starch colorimetry using commercial kits (Shanghai Enzyme-linked Biotechnology Co., Ltd., Shanghai, China) and expressed as enzyme activity per gram/microgram protein, respectively. The soluble protein content was determined by the Bradford method using commercial kit (Nanjing Jiancheng Bioengineering Institute, Nanjing, China).

#### 2.5.3. Plasma Biochemical Indexes

Plasma biochemical indicators including total protein (TP), total amino acid (TAA), and blood urea nitrogen (BUN) contents as well as aspartate transaminase (AST) and alanine transaminase (ALT) activities were determined by the BCA method, colorimetric method, urease method, colorimetric method, and colorimetric method using commercial assay kits (Nanjing Jiancheng Bioengineering Institute, Nanjing, China), respectively.

#### 2.5.4. Lysine and Protein Metabolism-Related Enzyme Activities

The activities of lysine *α*-ketoglutarate reductase (LKR), *α*-aminoadipate *δ*-semialdehyde synthase (AASS), glutamate dehydrogenase (GDH), and adenosine monophosphate deaminase (AMPD) in the liver and dorsal muscle were determined by the double antibody sandwich method using commercial ELISA kits (Shanghai Enzyme-linked Biotechnology Co., Ltd., Shanghai, China) and expressed as enzyme activity per gram/milligram protein.

#### 2.5.5. RNA Extraction, cDNA Synthesis, and Real-Time Quantitative PCR

According to the instructions, the TransZol Up Plus RNA kit was used to extract the total RNA in the liver. The RNA purity was tested with 1.2% agarose gel by an electrophoresis gel imaging system (Bio-Rad, America), and the RNA concentration was detected with a nucleic acid protein analyzer (NanoDrop 2000; Thermo, USA). Then, the total RNA of each group was used as the template for reverse transcription with Evo M-MLV reverse transcription kit II. The reaction system was 20 *μ*L: removal of genomic DNA products, 10 *μ*L; Evo M-MLV RTase Enzyme Mix, 1 *μ*L; RT Primer Mix, 1 *μ*L; 5x RTase Reaction Buffer Mix II, 4 *μ*L; and RNase Free dH_2_O, 4 *μ*L.

Using *β*-actin as internal reference, real-time quantitative PCR was performed using SYBR® Green Pro Taq HS Premix II qPCR kit and quantified on the LightCycler 480 (Roche Applied Science) using the following program: denaturation at 95°C for 30 s, followed by 40 cycles at 95°C of 5 s and 60°C for 30 s. The reaction system was 10 *μ*L: 2x SYBR® Green Premix Pro Taq HS Kit II, 5 *μ*L; 0.4 *μ*L for positive and negative primers, respectively; cDNA, 1 *μ*L; and RNase Free dH_2_O, 3.2 *μ*L. Primers of target genes used for qPCR are shown in Table 3. The relative gene expressions were calculated by the method of 2^-ΔΔCt^ [9].

### 2.6. Calculations and Statistical Analysis

The parameters were calculated using the following formulas:

(1) $$\begin{array}{c} \text{Feed intake }\left(\%\right)=100\times \frac{\text{feed consumption}}{\left(W_{i}+W_{f}\right)/\left(2\times d\right)},\\ \text{Weight gain rate }\left(\text{WGR},\%\right)=100\times \frac{W_{f}–W_{i}}{W_{i}},\\ \text{Specific growth rate }\left(\text{SGR},\%/\text{day}\right)=100\times \frac{\ln W_{f}–\ln W_{i}}{d},\\ \text{Feed conversion ratio }\left(\text{FCR}\right)=\frac{\text{feed consumption}}{W_{f}–W_{i}},\\ \text{Protein efficiency ratio }\left(\text{PER}\right)=\frac{W_{f}–W_{i}}{\text{protein intake}},\\ \text{Condition factor }\left(\text{CF},\text{g}/\text{c}{\text{m}}^{3}\right)=\frac{\text{body weight }\left(\text{g}\right)}{\text{body length }{\left(\text{cm}\right)}^{3}},\\ \text{Viscerosomatic index }\left(\text{VSI},\%\right)=100\times \frac{\text{viscerosomatic weight}\left(\text{g}\right)}{\text{whole body weight }\left(\text{g}\right)},\\ \text{Hepatosomatic index }\left(\text{HSI},\%\right)=100\times \frac{\text{hepatic weight}\left(\text{g}\right)}{\text{whole body weight }\left(\text{g}\right)},\\ \text{Intestine somatic index }\left(\text{ISI},\%\right)=100\times \frac{\text{intestine weight}\left(\text{g}\right)}{\text{whole body weight}\left(\text{g}\right)}, \end{array}$$

where *W*_*i*_, *W*_*f*_, and *d* stand for initial body weight (g), final body weight (g), and feeding day (d), respectively.

The Leaven and Kolmogorov-Smirnov tests were used to confirm the homoscedasticity assumptions and normality, respectively. All data (means ± SEM) were analyzed by a one-way analysis of variance (ANOVA) followed by Duncan's test to compare the means between individual treatments. The significant difference was set at the level of *P* < 0.05. For detecting potential linear or quadratic effects of dietary lysine level, all data were also subjected to polynomial orthogonal contrasts analysis. Statistical analysis was performed using SPSS 21.0 (SPSS Inc., Chicago, IL, USA) for Windows.

## 3. Results

### 3.1. Growth Performance and Body Indexes

The final body weight, WGR, and SGR were improved with the increasing dietary lysine level up to 2.30% and depressed thereafter (Table 4). Conversely, the FCR depressed with the increasing dietary lysine level up to 2.30% and increased afterward. Interestingly, CF enhanced linearly with the increasing dietary lysine level, which was significantly higher in the 3.56% and 4.36% lysine groups compared to the 1.10% lysine group (*P* < 0.05). The VSI was significantly higher in the 2.30%, 3.08%, and 3.56% lysine groups compared to the 1.69% lysine group (*P* < 0.05). However, the feed intake, PER, HSI, and ISI were not affected by dietary lysine level (*P* > 0.05).

Based on the second-order polynomial (SOP) model, the optimal lysine requirement for final body weight, WGR, SGR, and FCR of juvenile *P. leopardus* was estimated to be 2.70%, 2.68%, 2.70%, and 2.60% of diets, respectively.

### 3.2. Intestinal Digestive Enzyme Activities

The activities of intestinal trypsin and amylase were increased linearly with the increasing dietary lysine level, and those were significantly higher in the 3.08%, 3.56%, and 4.36% lysine groups compared to the 1.10% lysine group (*P* < 0.05; Table 5). However, the intestinal lipase activity improved with the increasing dietary lysine level up to 2.30% and decreased thereafter.

### 3.3. Plasma Biochemical Indexes

The plasma TP content was enhanced with the increasing dietary lysine level up to 1.69% and plateaued thereafter (Table 6). Conversely, the BUN content significantly depressed with the increasing dietary lysine level up to 2.30% and increased afterward. However, no significant differences were observed in the plasma TAA content, AST, and ALT activities as well as AST/ALT ratio among the treatment groups (*P* > 0.05).

### 3.4. Lysine Metabolism-Related Enzyme Activities

The hepatic LKR activity was significantly enhanced with the increasing dietary lysine level up to 1.69% and remained constant in the 2.30% and 3.08% lysine groups and then significantly reduced in the 3.56% and 4.36% lysine groups (*P* < 0.05; Table 7). No significant differences were found in the activities of LKR in muscle as well as AASS in the liver and muscle among the dietary treatments (*P* > 0.05).

### 3.5. Protein Metabolism-Related Enzyme Activities

The hepatic AMPD activity was generally reduced with the increasing dietary lysine level, which was significantly higher in the 1.10% lysine group compared to the other groups (*P* < 0.05; Table 8). However, no significant differences were found in the activities of GDH in the liver as well as GDH and AMPD in the muscle among the treatment groups (*P* > 0.05).

### 3.6. Relative Expression Levels of Mammalian Target of Rapamycin (mTOR) and Amino Acid Response (AAR) Pathway-Related Gene in the Liver

**Figure 1:**
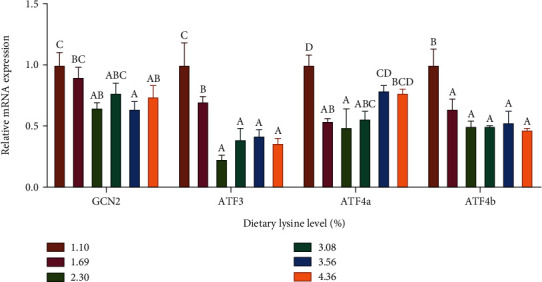
Relative expression levels of mammalian target of rapamycin signaling pathway including the target of rapamycin (TOR), S6 kinase 1 (S6K1), and eIF4E-binding protein 2 (4E-BP2) in the liver of *Plectropomus leopardus* fed diets with various levels of lysine. Values are means with standard errors represented by vertical bars (*n* = 6). ^A,B,C,D^Means with different letters were significantly different (*P* < 0.05).

The relative expression level of hepatic TOR was upregulated with the increasing dietary lysine level up to 1.69% and plateaued thereafter, which was significantly lower in the 1.10% lysine group compared to the 1.69%, 2.30%, and 4.36% lysine groups (*P* < 0.05; Figure 1). The relative expression level of hepatic 4E-BP2 was downregulated with the increasing dietary lysine level up to 1.69% and then upregulated thereafter. Conversely, the relative expression of hepatic S6K1 was upregulated with the increasing dietary lysine level up to 1.69% and then downregulated afterward, which was significantly higher in the 1.69% lysine group compared to the 1.10%, 2.30%, 3.56%, and 4.36% lysine groups (*P* < 0.05).

**Figure 2:**
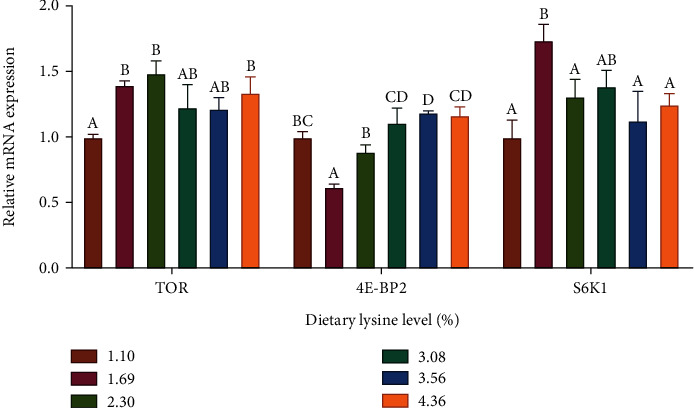
Relative expression levels of amino acid response signaling pathway including the general control nondepressible 2 (GCN2), activating transcription factor 3 (ATF3), activating transcription factor 4a (ATF4a), and activating transcription factor 4b (ATF4b) in the liver of *Plectropomus leopardus* fed diets with various levels of lysine. Values are means with standard errors represented by vertical bars (*n* = 6). ^A,B,C^Means with different letters were significantly different (*P* < 0.05).

The relative expression levels of GCN2, ATF3, and ATF4b in the liver were generally downregulated with the increasing dietary lysine level (Figure 2); thereinto, the relative expression level of GCN2 was significantly higher in the 1.10% lysine group compared to the 2.3%, 3.56%, and 4.36% lysine groups, and the relative expression levels of ATF3 and ATF4b were significantly higher in the 1.10% lysine group compared to the other groups (*P* < 0.05). The relative expression level of ATF4a was downregulated with the increasing dietary lysine level up to 2.30% and then upregulated thereafter, which was significantly lower in the 1.69%, 2.30%, and 3.08% lysine groups compared to the 1.10% lysine group (*P* < 0.05).

### 3.7. Whole-Body Composition

The whole-body crude protein content was generally enhanced with the increasing dietary lysine level up to 3.08% and declined afterward (Table 9). However, the whole-body crude lipid content was significantly higher in the 1.10% lysine group compared to the other groups (*P* < 0.05). No significant differences were found in the whole-body moisture and crude ash contents among the treatment groups (*P* > 0.05).

**Figure 3:**
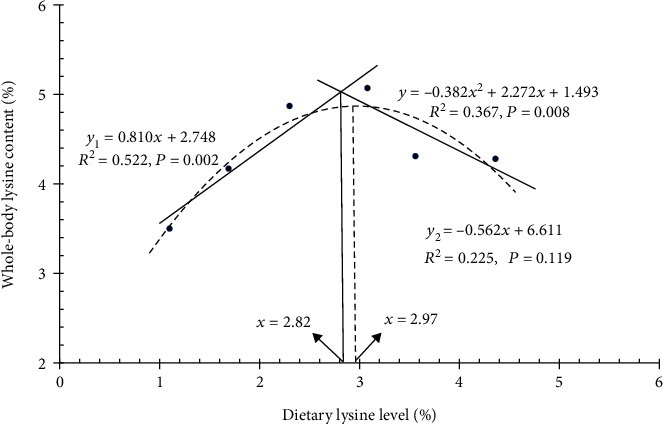
Broken-line (solid line) and second-order polynomial (dash line) regression analysis for optimization of dietary lysine content in relation to the whole-body lysine content in juvenile *Plectropomus leopardus*.

The whole-body lysine, arginine, isoleucine, leucine, aspartate, alanine, serine, and total amino acid contents were firstly increased and then decreased with the raising dietary lysine level, and the highest values were observed in the 3.08% lysine group (Table 9). Based on the broken-line and SOP regressions between the dietary lysine level and the whole-body lysine content, the optimal lysine requirement of juvenile *P. leopardus* was estimated to be 2.82% and 2.97% of diets, respectively (Figure 3).

## 4. Discussion

As one of the essential amino acids, lysine plays an important role in the normal growth and development of fish. The deficiency of lysine will lead to a decline in appetite and feed intake, which is not conducive to the growth and development of fish [10, 11]. However, excessive lysine also has negative effects on the growth and health of fish [5, 6] as it disrupts the balance of amino acids and prevents the absorption and utilization of other amino acids [7]. According to NRC [12], the lysine requirements of freshwater and marine fishes are 1.6–2.4% and 1.7–2.8%, respectively. In this study, dietary insufficient (1.10% and 1.69%) or excessive (3.56% and 4.36%) lysine retarded the growth rate and feed utilization, which reached the highest values in the 2.30–3.08% lysine groups. The lower growth rate of *P. leopardus* fed below the optimum dietary lysine may be due to the decline in lysine intake, while the depressed growth of fish fed diets with excessive lysine may be related to the lysine-arginine antagonism or the imbalanced amino acids. The dose-response trend is consistent with the finding for orange-spotted grouper (*Epinephelus coioides*) [13]. Based on the SOP regression model for growth rate and feed efficiency, the optimal dietary lysine requirement of *P. leopardus* was 2.60–2.68% of diet (4.91–5.06% of crude protein). The value was similar to that for juvenile orange-spotted grouper (2.83–3.04% of diet) [13, 14]. Nevertheless, it was higher than that for juvenile hybrid grouper (*Epinephelus fuscoguttatus♀× E. lanceolatus♂*) (2.16% of diet) [15], but lower than that for juvenile humpback grouper (*Cromileptes altivelis*) (3.99% of diet) [16]. Additionally, the previous study also showed that the dietary lysine requirements based on SGR were recommended as 3.04% and 2.61% of diets for juvenile (initial body weight 22.07 g) and subadult (initial body weight 102.51 g) orange-spotted grouper, respectively [13]. Consequently, the differences in dietary lysine requirements among these studies are possibly due to the species and size of groupers [17].

Additionally, body index (CF, VSI, HSI, and ISI) is also an important parameter reflecting the growth and health of fish. In this study, the CF was linearly increased with the raising dietary lysine level, which indicates that the increased dietary lysine level resulted in the elevation of obesity degree. The positive correlation between CF and dietary lysine level was also observed in silver perch (*Bidyanus bidyanus*) [18], Chinese sucker (*Myxocyprinus asiaticus*) [19], dusky kob (*Argyrosomus japonicus*) [20], and *Pseudobagrus ussuriensis* [21]. Additionally, the present and previous studies [14] showed that fish fed diets with optimal lysine exhibited higher VSI and whole-body protein content, which indicates that the balanced amino acids promoted the protein deposition of visceral organ.

As is known to all, digestive enzymes play a key role in the digestibility and utilization of nutrients [22]. A previous study with Jian carp (*Cyprinus carpio* var. *Jian*) showed that the activities of intestinal protease and lipase significantly improved with the increase of dietary lysine level [23]. A similar observation was found in this study, and the activities of intestinal trypsin and amylase linearly were enhanced with the increasing dietary lysine level. The positive effects of dietary lysine on the intestinal digestive enzymes' activities could be explained by different assumptions: lysine has the ability to stimulate the synthesis of these enzymes in the pancreas and/or lysine might affect the secretion and release of these enzymes in fish [24]. Additionally, the optimum dietary lysine significantly improved the intestinal lipase activity of *P. leopardus*, which was consistent with the results in large yellow croaker (*Pseudosciaena crocea*) [25] and grass carp (*Ctenopharyngodon idellus*) [26]. However, the intestinal lipase activity was depressed by excessive dietary lysine, indicating the digestive function of *P. leopardus* was adversely affected by the imbalance of dietary amino acids. Previous studies have also confirmed that excessive lysine level might inhibit lipase activity [24, 27]. Hence, appropriate dietary lysine enhanced digestive enzyme activities and thereby might improve the digestion and growth of *P. leopardus*.

Protein metabolism is a dynamic process involving the balance between the synthesis and degradation of protein [28]. It is well known that all amino acids are needed for protein synthesis, and a deficiency in one of these amino acids may diminish the protein synthetic response [29]. The TOR is a serine/threonine kinase, which regulates protein synthesis [30]. There are two structurally and functionally distinct TOR complexes (TORC). TORC1 is sensitive to rapamycin and nutrients, while TORC2 is insensitive [31, 32]. After TORC1 was activated, the downstream main-acting factors 4EBPs and S6K were phosphorylated to activate protein translation [33–35]. In this study, dietary lysine inclusion of 1.69–2.30% upregulated the relative expression levels of TOR and S6K1 but downregulated the relative expression level of 4E-BP2, indicating that the optimum dietary lysine activated the mTOR signaling pathway and thereby promoted the protein synthesis in *P. leopardus*. Similarly, the optimum dietary lysine significantly upregulated the relative expression levels of hepatic TOR in juvenile *Hemibagrus wyckioides* [36] and hepatic TOR and S6K1 in hybrid grouper [15] and upregulated the relative expression level of intestinal TOR but downregulated the relative expression level of intestinal 4E-BP in grass carp [26]. Therefore, it is believed that a balanced dietary amino acid profile plays an important role in sustaining efficient protein synthesis [37].

On the other hand, ammonia nitrogen is a product of protein catabolism, which reflects the ability to use amino acids as energy source. In this study, optimal dietary lysine inclusion increased the plasma TP content but depressed the BUN content, indicating that protein utilization was improved in *P. leopardus* fed diets with 2.30–3.08% lysine. A similar observation was found in a previous study with dace (*Leuciscus brandti*) [38]. Lysine is converted to saccharopine in the liver mitochondria, and the catabolism of saccharopine is catalyzed by LKR and saccharopine dehydrogenase [39]. AASS is a bifunctional enzyme composed of both LKR and saccharopine dehydrogenase domains [40, 41]. When dietary lysine is limited, the degradation of lysine is reduced so that lysine can be preserved in protein synthesis [42]. Accordingly, the hepatic LKR and AASS activities in dace were significantly increased with the increasing dietary lysine level [38]. A similar result was observed in this study, and the hepatic LKR activity was significantly increased by dietary 1.69%–3.08% lysine inclusion. However, the activity and mRNA level of hepatic LKR in rainbow trout (*Oncorhynchus mykiss*) were not affected by dietary lysine level [43, 44]. GDH and AMPD are the key rate-limiting enzymes in the combined amino acid deamination reaction [45], which are usually considered evaluation indicators for using amino acids as an energy source [46]. In this study, the hepatic AMPD activity was linearly depressed with the increase of dietary lysine level, indicating that dietary lysine inclusion was conducive to reducing protein catabolism and thereby improving protein deposition. In the study of juvenile *Hemibagrus wyckioides*, optimal lysine also downregulated the relative expression levels of GDH in the liver and AMPD in the liver and muscle [36]. Additionally, dietary protein or amino acid deficiency will trigger a series of signal processes, collectively referred to as AAR [47, 48]. GCN2 is an essential regulator of cellular response to amino acid limitation in all tissues [35]. When GCN2 kinase is activated in the absence of amino acids, its downstream ATF3 and ATF4 are also activated, thus inhibiting the initiation of the translation of protein in cells and reducing overall protein synthesis [49]. The present study showed that the relative expression levels of GCN2, ATF3, ATF4a, and ATF4b in the liver of *P. leopardus* were downregulated by dietary inclusion of 2.30% lysine, suggesting that dietary suitable lysine level and balanced amino acids inhibited the AAR signaling pathway and were more conducive to protein synthesis.

Appropriate dietary lysine level is conducive to the utilization of amino acids and the synthesis of body protein. The present study showed that dietary 3.08% lysine improved the whole-body crude protein content but depressed the whole-body lipid content of juvenile *P. leopardus*. Similarly, previous studies also showed that appropriate dietary lysine reduced body lipid content but increased body protein content of channel catfish (*Ictalurus punctatus*) [50], rainbow trout [51, 52], Japanese seabass (*Lateolabrax japonicus*) [53], large yellow croaker [54], silver perch [18], and tilapia (*Oreochromis niloticus*) [55]. Meanwhile, lysine is also a precursor of carnitine, and carnitine involves in the entry of long-chain fatty acyl groups into the mitochondria for *β*-oxidation [43]. Hence, the lower protein content and higher lipid content in fish fed diets with scarce lysine may be explained by reduced *β*-oxidation of fatty acids, resulting in more utilization of protein rather than lipid as an energy source [56, 57]. Additionally, the changing trend of the whole-body amino acid composition is consistent with the crude protein content. The contents of lysine, arginine, isoleucine, leucine, aspartate, alanine, and serine as well as total amino acids increased with the increasing dietary lysine level up to 3.08% and declined or remained constant afterward. A balanced dietary amino acid may increase the retention of amino acids, thus increasing protein retention. The lack and excess of dietary lysine may increase the consumption of amino acids as energy while decreasing the amino acids used for protein synthesis [37]. Likewise, Mai et al. [53] found that dietary lysine levels significantly impacted the amino acid contents in the muscle of Japanese bass, which was corresponding to the results of tiger puffer [55]. In the study with tilapia, the whole-body essential amino acid compositions were not changed with various dietary lysine levels, whereas the nonessential amino acid contents were increased [56]. However, some studies have shown that dietary lysine level had no effects on the whole-body composition [13, 58, 59] and amino acid profile [59, 60] of fish. The discrepancy is possibly due to the differences in the feed composition, sensitivity, and tolerance to amino acids of different fish species.

## 5. Conclusion

Based on the SOP regression model, the optimal dietary lysine requirement of *P. leopardus* was 2.60%–2.97% of diets (4.91%–5.60% of diet protein) for growth performance, feed utilization, and lysine deposition. The optimal dietary lysine promoted the digestive enzymes' activities and protein synthesis, but depressed protein degradation thereby enhanced protein deposition and growth performance of juvenile *P. leopardus*. However, deficiency and excess dietary lysine resulted in amino acid imbalance and thereby repressed the growth of juvenile *P. leopardus*.

## Figures and Tables

**Table 1:** Formulation and proximate composition (% dry matter) of the experimental diets.

	Dietary lysine level (%)					
	1.10	1.69	2.30	3.08	3.56	4.36
Ingredient						
Fish meal	18.00	18.00	18.00	18.00	18.00	18.00
Corn gluten meal	15.00	15.00	15.00	15.00	15.00	15.00
Coated L-lysine (69.86%)^1^	0.00	0.86	1.72	2.58	3.43	4.29
Coated L-alanine (69.93%)^1^	4.29	3.43	2.58	1.72	0.86	0.00
Amino acid mixture^2^	25.72	25.72	25.72	25.72	25.72	25.72
Guar gum	2.00	2.00	2.00	2.00	2.00	2.00
Fish oil	4.70	4.70	4.70	4.70	4.70	4.70
Soybean oil	3.65	3.65	3.65	3.65	3.65	3.65
Soybean phospholipid	2.00	2.00	2.00	2.00	2.00	2.00
Wheat flour	21.67	21.67	21.67	21.67	21.67	21.67
Ca(H_2_PO_4_)_2_	1.00	1.00	1.00	1.00	1.00	1.00
Choline chloride	0.40	0.40	0.40	0.40	0.40	0.40
Vitamin C	0.03	0.03	0.03	0.03	0.03	0.03
Ethoxyquin	0.02	0.02	0.02	0.02	0.02	0.02
Y_2_O_3_	0.02	0.02	0.02	0.02	0.02	0.02
Vitamin premix^3^	0.50	0.50	0.50	0.50	0.50	0.50
Mineral premix^3^	1.00	1.00	1.00	1.00	1.00	1.00
Proximate composition						
Dry matter (DM, %)	92.73	92.86	92.67	92.57	92.49	92.24
Crude protein (% DM)	53.01	53.30	53.06	52.59	52.28	53.09
Crude lipid (% DM)	11.93	12.76	12.88	12.83	12.64	12.63
Crude ash (% DM)	5.90	5.74	4.66	4.59	4.65	4.62

^1^Supplied by the Shaanxi Baichuan Biotechnology Co., Ltd., Xi'an, China. ^2^Amino acid mixture included 1.02% DL-methionine, 1.60% L-threonine, 2.52% L-arginine, 1.66% L-isoleucine, 1.97% L-leucine, 1.47% L-valine, 0.59% L-histidine, 1.18% L-phenylalanine, 0.25% L-cystine, 1.83% L-glycine, 1.03% L-serine, 1.53% L-alanine, 4.10% L-aspartate, 3.90% L-glutamate, and 1.07% L-tyrosine. ^3^Supplied by the Qingdao Master Bio-Tech Co., Ltd., Qingdao, China. Vitamin premix (g/kg of mixture) included 1.55 g retinyl acetate (2 800 000 IU/g), 0.03 g cholecalciferol, 40 g DL-*α*-tocopheryl acetate, 8 g menadione, 6 g thiamine hydrochloride, 9 g riboflavin, 7 g pyridoxine hydrochloride, 0.05 g vitamin B_12_, 50 g ascorbic acid, 30 g calcium D-pantothenate, 45 g niacin, 2.5 g folic acid, 0.1 g D-biotin, and 100 g inositol. Mineral premix (g/kg of mixture) included 186 g FeSO_4_·7H_2_O, 53 g ZnSO_4_·7H_2_O, 25 g MnSO_4_·H_2_O, 2.7 g CuSO_4_·5H_2_O, 0.4 g CoCl_2_·6H_2_O, 0.1 g Na_2_SeO_3_, and 0.13 g KI.

**Table 2:** Amino acid profile of the experimental diets (% dry matter).

	Dietary lysine level (%)					
	1.10	1.69	2.30	3.08	3.56	4.36
Essential amino acids (EAA)						
Methionine	1.37	1.37	1.37	1.29	1.37	1.46
Lysine	1.10	1.69	2.30	3.08	3.56	4.36
Threonine	2.45	2.37	2.37	2.46	2.39	2.44
Isoleucine	2.35	2.37	2.35	2.30	2.30	2.40
Leucine	4.31	4.28	4.29	4.36	4.31	4.32
Phenylalanine	2.26	2.25	2.23	2.30	2.26	2.28
Arginine	3.33	3.19	3.25	3.06	2.91	3.03
Histidine	1.14	1.15	1.12	1.18	1.14	1.16
Valine	2.49	2.47	2.48	2.74	2.71	2.88
∑EAA	20.81	21.13	21.79	22.78	22.95	24.34
Nonessential amino acids (NEAA)						
Aspartate	5.83	5.78	5.77	5.88	5.76	5.84
Glutamate	8.13	8.01	8.07	8.30	8.15	8.32
Glycine	2.73	2.74	2.70	2.78	2.70	2.79
Alanine	7.04	6.31	5.55	4.67	3.86	3.11
Cystine	0.65	0.61	0.65	0.66	0.64	0.57
Serine	1.97	1.94	1.91	1.98	1.99	1.99
Proline	1.77	1.76	1.65	1.84	1.83	1.66
Tyrosine	1.96	1.92	1.91	1.94	1.92	1.95
∑NEAA	30.09	29.06	28.22	28.03	26.85	26.23

Tryptophan was not determined.

**Table 3:** The forward and reverse primers used for real-time quantitative PCR analysis and accession numbers of gene sequences (GenBank).

Primers	Forward and reverse primer sequence (5′-3′)	GenBank accession no.
TOR	F: CAAGGTTTCTTCCGCTCCATCTCC R: CTCCACCAGGGCTTCATTCACTTC	JN850959.1
S6K1	F: GTGAAAGGGAGGAGCTTGGC R: AACACGAGACTGCTGAGGGT	XM_033643204.1
4EBP2	F: CAAGAAGAACGAAGCCAACAACCAC R: GTGTCGCTCCAGTCTGTTAGATGTC	XM_033613907.1
GCN2	F: AGGAGGACTGTCTCGTGGTGAAC R: GAGTGTGGTTGGTGAGGCTTTGG	XM_042500953.1
ATF3	F: CCAAACACCCGAGGATGAGAGAAAC R: GGAAGTGGAGGTGGTGGAGGAG	XM_042503803.1
ATF4a	F: TGGAGCAGACGATGGCAAAGATG R: CGGATGAGCAGGAACCAATGAGG	XM_042485343.1
ATF4b	F: GGATCTGAGCGAGTTGGACATTGAG R: GAGGCGAGGAGGTCTTCTGGAG	XM_042505784.1
*β*-Actin	F: TGAGAGGTTCCGTTGCCCAGAG R: CTGTTGTAGGTGGTCTCGTGGATTC	AY510710.2

TOR = target of rapamycin; S6K1 = S6 kinase 1; 4EBP2 = eIF4E-binding protein 2; GCN2 = general control nondepressible 2; ATF3 = activating transcription factor 3; ATF4a = activating transcription factor 4a; ATF4b = activating transcription factor 4b.

**Table 4:** Growth performance, feed utilization, and body indexes of juvenile *Plectropomus leopardus* fed diets with various levels of lysine.

Dietary lysine level (%)	Final weight (g)	Feed intake (%)	WGR (%)	SGR (%/d)	Survival rate (%)	FCR	PER	CF (g/cm^3^)	VSI (%)	HSI (%)	ISI (%)
1.10	17.02 ± 0.44^a^	2.36 ± 0.13	61.11 ± 4.06^a^	0.85 ± 0.04^a^	80.00 ± 2.31	3.97 ± 0.71^bc^	0.76 ± 0.11	1.67 ± 0.02^a^	4.03 ± 0.13^ab^	0.99 ± 0.02	0.88 ± 0.04
1.69	20.13 ± 0.89^b^	2.34 ± 0.11	90.48 ± 8.20^bc^	1.15 ± 0.08^c^	74.67 ± 3.53	2.99 ± 0.17^ab^	1.15 ± 0.11	1.75 ± 0.03^ab^	3.94 ± 0.19^a^	1.02 ± 0.14	0.86 ± 0.08
2.30	21.01 ± 1.01^c^	2.31 ± 0.10	98.59 ± 16.71^c^	1.22 ± 0.09^c^	84.00 ± 4.00	2.38 ± 0.33^a^	1.20 ± 0.13	1.77 ± 0.02^bc^	4.86 ± 0.04^b^	0.99 ± 0.10	0.91 ± 0.07
3.08	20.29 ± 1.40^c^	2.47 ± 0.05	91.97 ± 13.17^c^	1.15 ± 0.05^c^	76.00 ± 2.31	3.04 ± 0.36^ab^	1.11 ± 0.17	1.81 ± 0.02^bc^	4.64 ± 0.08^b^	1.01 ± 0.02	1.01 ± 0.05
3.56	19.70 ± 0.48^abc^	2.45 ± 0.06	86.25 ± 4.56^abc^	1.11 ± 0.08^bc^	80.00 ± 4.00	2.90 ± 0.23^ab^	1.04 ± 0.05	1.82 ± 0.01^c^	4.58 ± 0.07^b^	0.93 ± 0.10	0.98 ± 0.03
4.36	17.39 ± 0.52^ab^	2.63 ± 0.05	64.39 ± 4.75^ab^	0.88 ± 0.09^ab^	78.67 ± 1.33	4.43 ± 0.26^c^	0.77 ± 0.06	1.89 ± 0.02^d^	4.02 ± 0.18^ab^	0.90 ± 0.06	0.91 ± 0.05
ANOVA											
*P* value	0.031	0.209	0.030	0.019	0.385	0.048	0.059	<0.001	<0.001	0.857	0.393
Regression											
Model	SOP	L	SOP	SOP	NR	SOP	SOP	L	SOP	NR	NR
*P* value	0.002	0.018	0.002	0.001	0.994	0.004	0.006	<0.001	<0.001	0.247	0.211
Adjusted *R*^2^	0.505	0.257	0.508	0.541	0.001	0.461	0.431	0.592	0.379	0.025	0.022
OIDL	2.70	/	2.68	2.70	/	2.60	2.64	/	2.83	/	/

Values are means ± standard error (SE) of three replications. Means with different superscript letter(s) in the same row are significantly different (*P* < 0.05). WGR = weight gain rate; SGR = specific growth rate; FCR = feed conversion ratio; PER = protein efficiency ratio; CF = condition factor; VSI = viscerosomatic index; HSI = hepatosomatic index; ISI = intestine somatic index; SOP = second-order polynomial trend; L = linear trend; NR = no relationship; OIDL = optimal inclusion of dietary lysine level.

**Table 5:** Intestinal digestive enzyme activities of juvenile *Plectropomus leopardus* fed diets with various levels of lysine.

Dietary lysine level (%)	Trypsin (U/*μ*g protein)	Amylase (IU/mg protein)	Lipase (U/g protein)
1.10	3.98 ± 0.13^a^	0.19 ± 0.01^a^	7.02 ± 0.29^a^
1.69	5.67 ± 0.32^bc^	0.19 ± 0.03^a^	8.62 ± 0.70^ab^
2.30	5.28 ± 0.09^b^	0.22 ± 0.03^ab^	10.75 ± 0.67^c^
3.08	5.53 ± 0.16^bc^	0.28 ± 0.03^b^	9.85 ± 0.41^bc^
3.56	5.85 ± 0.02^bc^	0.29 ± 0.03^b^	9.66 ± 0.50^bc^
4.36	5.89 ± 0.23^c^	0.30 ± 0.03^b^	8.56 ± 0.52^ab^
ANOVA			
*P* value	<0.001	0.016	0.002
Regression			
Model	L	L	SOP
*P* value	<0.001	<0.001	<0.001
Adjusted *R*^2^	0.438	0.373	0.501
OIDL	/	/	2.94

Values are means ± standard error (SE) of three replications. Means with different superscript letter(s) in the same row are significantly different (*P* < 0.05). SOP = second-order polynomial trend; L = linear trend; OIDL = optimal inclusion of dietary lysine level.

**Table 6:** Biochemical parameters in plasma of *Plectropomus leopardus* fed diets with various levels of lysine.

Dietary lysine level (%)	TP (*μ*g/mL)	TAA (mmol/L)	BUN (*μ*mol/mL)	AST (U/*μ*L)	ALT (U/*μ*L)	AST/ALT
1.10	2.09 ± 0.18^a^	0.86 ± 0.06	127.50 ± 5.18^d^	16.77 ± 1.17	7.27 ± 1.25	2.48 ± 0.51
1.69	3.13 ± 0.18^b^	0.99 ± 0.14	32.99 ± 0.97^c^	15.38 ± 4.07	7.25 ± 0.42	2.20 ± 0.72
2.30	3.34 ± 0.08^b^	0.94 ± 0.18	5.79 ± 1.51^a^	15.95 ± 0.46	5.51 ± 0.72	2.97 ± 0.28
3.08	3.09 ± 0.23^b^	0.96 ± 0.05	6.34 ± 0.32^a^	19.55 ± 1.80	5.20 ± 0.87	4.06 ± 0.89
3.56	3.25 ± 0.08^b^	0.81 ± 0.05	18.78 ± 3.43^b^	17.71 ± 0.99	6.45 ± 0.27	2.77 ± 0.27
4.36	3.42 ± 0.07^b^	0.74 ± 0.08	22.01 ± 1.85^b^	12.10 ± 1.42	6.77 ± 0.42	1.83 ± 0.33
ANOVA						
*P* value	0.001	0.193	<0.001	0.249	0.289	0.159
Regression						
Model	L	SOP	SOP	NR	NR	NR
*P* value	0.003	0.043	<0.001	0.203	0.119	0.119
Adjusted *R*^2^	0.390	0.123	0.839	0.084	0.147	0.146
OIDL	/	2.24	3.17	/	/	/

Values are means ± standard error (SE) of three replications. Means with different superscript letter(s) in the same row are significantly different (*P* < 0.05). TP = total protein; TAA = total amino acids; BUN = blood urea nitrogen; AST = aspartate aminotransferase; ALT = alanine aminotransferase; SOP = second-order polynomial trend; NR = no relationship; OIDL = optimal inclusion of dietary lysine level.

**Table 7:** Lysine metabolism-related parameters in the liver and muscle of juvenile *Plectropomus leopardus* fed diets with various levels of lysine.

Dietary lysine level (%)	Liver		Muscle	
	LKR (U/g protein)	AASS (U/mg protein)	LKR (U/g protein)	AASS (U/mg protein)
1.10	8.78 ± 2.06^a^	0.29 ± 0.04	30.52 ± 0.75	0.37 ± 0.03
1.69	17.15 ± 1.34^b^	0.27 ± 0.02	27.38 ± 0.68	0.32 ± 0.02
2.30	16.84 ± 0.14^b^	0.32 ± 0.03	29.68 ± 2.06	0.32 ± 0.03
3.08	14.62 ± 0.74^b^	0.27 ± 0.02	31.20 ± 3.28	0.36 ± 0.04
3.56	9.89 ± 0.62^a^	0.28 ± 0.05	27.39 ± 1.38	0.32 ± 0.02
4.36	9.77 ± 1.63^a^	0.19 ± 0.03	32.49 ± 1.87	0.30 ± 0.02
ANOVA				
*P* value	0.001	0.168	0.385	0.333
Regression				
Model	SOP	SOP	NR	NR
*P* value	0.007	0.041	0.062	0.168
Adjusted *R*^2^	0.419	0.127	0.150	0.028
OIDL	2.20	2.12	/	/

Values are means ± standard error (SE) of three replications. Means with different superscript letter(s) in the same row are significantly different (*P* < 0.05). LKR = *α*-ketoglutarate reductase; AASS = *α*-aminoadipate-*γ*-semialdehyde; SOP = second-order polynomial trend; NR = no relationship; OIDL = optimal inclusion of dietary lysine level.

**Table 8:** Protein metabolism-related parameters in the liver and muscle of juvenile *Plectropomus leopardus* fed diets with various levels of lysine.

Dietary lysine level (%)	Liver		Muscle	
	GDH (U/mg protein)	AMPD (U/g protein)	GDH (U/g protein)	AMPD (U/g protein)
1.10	0.11 ± 0.01	97.86 ± 6.28^b^	0.71 ± 0.02	0.30 ± 0.01
1.69	0.12 ± 0.01	73.41 ± 8.09^a^	0.67 ± 0.05	0.28 ± 0.02
2.30	0.11 ± 0.00	71.99 ± 1.33^a^	0.68 ± 0.05	0.31 ± 0.01
3.08	0.11 ± 0.00	72.50 ± 1.00^a^	0.69 ± 0.07	0.28 ± 0.03
3.56	0.11 ± 0.00	71.46 ± 3.17^a^	0.75 ± 0.01	0.28 ± 0.01
4.36	0.11 ± 0.01	68.98 ± 8.73^a^	0.74 ± 0.02	0.27 ± 0.02
ANOVA				
*P* value	0.591	0.032	0.763	0.585
Regression				
Model	NR	L	NR	NR
*P* value	0.246	0.015	0.267	0.191
Adjusted *R*^2^	0.026	0.274	0.019	0.048
OIDL	/	/	/	/

Values are means ± standard error (SE) of three replications. Means with different superscript letter(s) in the same row are significantly different (*P* < 0.05). GDH = glutamate dehydrogenase; AMPD = adenosine monophosphate deaminase; NR = no relationship; SOP = second-order polynomial trend; OIDL = optimal inclusion of dietary lysine level.

**Table 9:** Proximate composition and amino acid composition of the whole body of *Plectropomus leopardus* fed diets with various levels of lysine.

	Dietary lysine level (%)					
	1.10	1.69	2.30	3.08	3.56	4.36
Proximate composition (wet weight basis, %)						
Moisture (%)	75.00 ± 0.08	76.10 ± 0.24	76.02 ± 0.47	75.56 ± 0.27	76.49 ± 1.02	75.82 ± 0.26
Crude protein (%)	13.93 ± 0.32^ab^	13.24 ± 0.34^a^	14.46 ± 0.53^b^	16.48 ± 0.35^c^	14.38 ± 0.11^ab^	15.00 ± 0.34^b^
Crude lipid (%)	1.97 ± 0.07^b^	1.66 ± 0.15^a^	1.57 ± 0.12^a^	1.53 ± 0.09^a^	1.48 ± 0.09^a^	1.52 ± 0.10^a^
Crude ash (%)	6.94 ± 1.18	6.75 ± 1.02	6.11 ± 0.85	6.41 ± 0.62	6.82 ± 1.28	6.65 ± 0.64
Amino acid composition (dry matter basis, %)						
Methionine+cystine	1.37 ± 0.16	1.46 ± 0.10	1.72 ± 0.09	1.72 ± 0.13	1.51 ± 0.13	1.41 ± 0.12
Lysine	3.50 ± 0.57^a^	4.17 ± 0.20^ab^	4.87 ± 0.12^b^	5.07 ± 0.13^b^	4.31 ± 0.44^ab^	4.28 ± 0.22^ab^
Arginine	3.72 ± 0.21^a^	3.59 ± 0.05^a^	3.82 ± 0.03^a^	4.47 ± 0.06^b^	3.56 ± 004^a^	3.49 ± 0.17^a^
Threonine	2.10 ± 0.26	2.16 ± 0.20	2.39 ± 0.21	2.42 ± 0.26	2.14 ± 0.28	2.05 ± 0.26
Isoleucine	1.63 ± 0.26^a^	1.99 ± 0.11^ab^	2.29 ± 0.05^b^	2.40 ± 0.05^b^	2.08 ± 0.19^ab^	2.02 ± 0.12^ab^
Leucine	3.06 ± 0.44^a^	3.32 ± 0.21^a^	4.19 ± 0.06^b^	4.29 ± 0.08^b^	2.78 ± 0.26^a^	2.96 ± 0.33^a^
Phenylalanine	1.95 ± 0.25	2.01 ± 0.19	2.18 ± 0.23	2.28 ± 0.27	1.97 ± 0.29	1.91 ± 0.27
Histidine	0.89 ± 0.12	0.96 ± 0.06	1.05 ± 0.11	1.08 ± 0.10	0.92 ± 0.12	0.97 ± 0.12
Valine	2.00 ± 0.26	2.04 ± 0.21	2.28 ± 0.20	2.30 ± 0.30	2.04 ± 0.32	1.94 ± 0.27
Aspartate	4.64 ± 0.58^a^	5.30 ± 0.25^ab^	6.00 ± 0.14^b^	6.31 ± 0.16^b^	5.43 ± 0.39^ab^	5.33 ± 0.17^ab^
Glutamate	7.01 ± 0.85	7.34 ± 0.63	8.03 ± 0.74	8.18 ± 0.96	7.20 ± 0.97	7.13 ± 0.93
Glycine	6.15 ± 0.25	5.95 ± 0.55	5.44 ± 0.31	6.60 ± 0.19	5.82 ± 0.49	6.12 ± 0.73
Alanine	4.17 ± 0.17^a^	4.55 ± 0.12^ab^	4.53 ± 0.07^ab^	4.93 ± 0.15^b^	4.22 ± 0.14^a^	4.21 ± 0.19^a^
Serine	2.02 ± 0.19^a^	2.08 ± 0.10^a^	2.56 ± 0.02^b^	2.62 ± 0.05^b^	2.32 ± 0.12^ab^	2.29 ± 0.06^ab^
Proline	3.33 ± 0.12	3.36 ± 0.25	3.22 ± 0.22	3.80 ± 0.08	3.44 ± 0.28	3.28 ± 0.31
Tyrosine	1.48 ± 0.19	1.61 ± 0.14	1.82 ± 0.18	1.81 ± 0.17	1.57 ± 0.25	1.60 ± 0.23
∑Amino acids	48.99 ± 2.98^a^	53.02 ± 2.47^ab^	57.82 ± 0.79^bc^	62.43 ± 1.19^c^	53.81 ± 3.26^ab^	54.93 ± 1.18^ab^

Values are means ± standard error (SE) of three replications. Means with different superscript letter(s) in the same row are significantly different (*P* < 0.05). Tryptophan was not determined.

## Data Availability

The data used to support the findings of this study are available from the corresponding authors upon request.
